# Capturing chloroplast variation for molecular ecology studies: a simple next generation sequencing approach applied to a rainforest tree

**DOI:** 10.1186/1472-6785-13-8

**Published:** 2013-03-14

**Authors:** Hannah McPherson, Marlien van der Merwe, Sven K Delaney, Mark A Edwards, Robert J Henry, Emma McIntosh, Paul D Rymer, Melita L Milner, Juelian Siow, Maurizio Rossetto

**Affiliations:** 1 National Herbarium of NSW, The Royal Botanic Gardens and Domain Trust, Mrs Macquaries Road, 2000, Sydney, NSW, Australia; 2 Australian Centre for Evolutionary Biology and Biodiversity, School of Earth and Environmental Science, University of Adelaide, Adelaide, SA, Australia; 3 School of Biotechnology and Biomolecular Sciences, University of New South Wales, 2052, Sydney, NSW, Australia; 4 Southern Cross Plant Science, Southern Cross University, 2480, Lismore, NSW, Australia; 5 Queensland Alliance for Agriculture and Food Innovation, University of Queensland, 4072, Brisbane, QLD, Australia; 6 Hawkesbury Institute for the Environment, University of Western Sydney, Hawkesbury Campus, 2753, Richmond, NSW, Australia

**Keywords:** Illumina, Shotgun sequencing, Chloroplast genome, SNP, Phylogeography

## Abstract

**Background:**

With high quantity and quality data production and low cost, next generation sequencing has the potential to provide new opportunities for plant phylogeographic studies on single and multiple species. Here we present an approach for *in silicio* chloroplast DNA assembly and single nucleotide polymorphism detection from short-read shotgun sequencing. The approach is simple and effective and can be implemented using standard bioinformatic tools.

**Results:**

The chloroplast genome of *Toona ciliata* (Meliaceae), 159,514 base pairs long, was assembled from shotgun sequencing on the Illumina platform using *de novo* assembly of contigs. To evaluate its practicality, value and quality, we compared the short read assembly with an assembly completed using 454 data obtained after chloroplast DNA isolation. Sanger sequence verifications indicated that the Illumina dataset outperformed the longer read 454 data. Pooling of several individuals during preparation of the shotgun library enabled detection of informative chloroplast SNP markers. Following validation, we used the identified SNPs for a preliminary phylogeographic study of *T. ciliata* in Australia and to confirm low diversity across the distribution.

**Conclusions:**

Our approach provides a simple method for construction of whole chloroplast genomes from shotgun sequencing of whole genomic DNA using short-read data and no available closely related reference genome (e.g. from the same species or genus). The high coverage of Illumina sequence data also renders this method appropriate for multiplexing and SNP discovery and therefore a useful approach for landscape level studies of evolutionary ecology.

## Background

Landscape-scale studies of phylogenetic patterns and population dynamics across one or more species can reveal the genetic signatures of contrasting temporal responses to environmental change among diverse functional groups. Such information is critical for understanding how species are distributed, how communities are assembled, and in recognising different susceptibilities to threats and change [1].

The chloroplast genome provides an invaluable resource for studies of the evolution and molecular ecology of plants at a range of taxonomic levels. Because it is predominantly uniparentally inherited, has a low mutation rate, and does not recombine, it can be used to construct lineage genealogies that can be analysed in a spatial context [2,3]. For example, chloroplast DNA-based studies have revealed the postglacial recolonisation routes of many plants, including *Quercus*[4,5], *Fraxinus*[6], *Pinus*[7], and *Eucalyptus*[8].

A limitation of chloroplast markers is that the level of variation is often low compared with nuclear, or mitochondrial markers in animals, due to the slow rate of evolution of the chloroplast genome [3,9]. This is evident from the absence of a single widely used barcoding gene for plants [10]. Obtaining analytically informative data from the chloroplast DNA of non-model species can therefore be resource and time intensive, since preliminary investigations are often needed to identify sufficiently variable regions. Accordingly, multi-species studies can become prohibitively complex and expensive, an issue that is particularly relevant in high biodiversity systems such as rainforests.

Next generation sequencing (NGS) has ushered in a new era of opportunity for molecular ecology research. The low cost per base pair (bp), along with extremely high yield and the opportunity for multiplexing, means that investigating whole-chloroplast genomes, rather than targeting individual regions, is now a real possibility [11,12].

Most high-throughput sequencing of chloroplast genomes uses multiplexed PCR amplicons or enriched chloroplast DNA e.g. [13-17]. Optimisation of these methods for a range of species can be time consuming and costly. Cronn *et al.*[18] also present alternative techniques of plastid enrichment, e.g. exon capture, but note that for large scale studies including many individuals many of the reviewed reduction approaches can be expensive. In 2011 Nock *et al.*[11] accurately assembled the rice chloroplast genome from genomic DNA using 36 bp paired-end reads generated using the Illumina platform. The whole chloroplast sequence for *Oryza sativa japonica* (cultivar Nipponbare) was used as a reference to map the short reads. Assembly by read mapping was reliable enough to uncover a single nucleotide error in the reference sequence. Alignment with the reference sequence for domesticated rice was used to compare the chloroplast genome sequence of wild *Oryza* species in Asia and Australia [19]. However, in the absence of a closely related reference sequence, read mapping is not accurate or effective.

Recently, Zhang *et al.*[20] published a method for chloroplast genome assembly from whole genome data in the absence of a close reference sequence, using the 454 GS FLX (454) platform. To date, 454 has been the preferred platform for constructing chloroplast genomes without a closely related reference sequence because the longer reads make assembly easier and more reliable. Although the short reads of Illumina technology can be more difficult to assemble than 454 data, the greater depth of sequencing gives Illumina the lowest cost per base [21], making it an attractive option for multiplexing and SNP detection [14,22]. Isolation of chloroplast DNA requires significant additional effort, and the success of amplification will be sequence dependant. Analysis of a total DNA preparation rather than a chloroplast-enriched sample is a method with potential for much simpler and more widespread and routine applications.

Here we present an efficient approach for the assembly of non-model whole chloroplast genomes (i.e. those for which there is no closely related reference genome available) using shotgun sequencing of whole genomic DNA on the Solexa Illumina platform. The target species, red cedar (*Toona ciliata* M.Roem, Meliaceae), is a medium to large rainforest tree of economic and conservation significance, widespread along the east coast of Australia and across the Indo-Malesian archipelago into India [23]. The value of its timber has contributed towards a decline in the occurrence of large specimens in Australia. We selected red cedar for this study because of its economic importance and because of its distribution across a range of Australian rainforest habitats from the Sydney region to northern Australia. It is an early successional, prolific and highly dispersed tree species and potentially is among the rainforest taxa that recently arrived in Australia from northern origins [24]. Because of its likely recent expansion history, and the fact that past studies discovered low nuclear diversity [25,26], we expected to find low levels of chloroplast DNA diversity across its distribution and, as a result, we considered it an ideal target species for assessing the advantages of full chloroplast DNA shotgun sequencing.

## Methods

To carry out this study we generated three NGS datasets for *Toona ciliata*:

1. Illumina shotgun sequence data generated using pooled whole genomic DNA from four individuals collected in the Big Scrub at Nightcap National Park (New South Wales, Australia).

2. 454 data from chloroplast-enriched DNA for a single individual collected in the Royal Botanic Gardens, Sydney (New South Wales, Australia).

3. Illumina shotgun sequence data generated using pooled whole genomic DNA from four individuals collected in Royal National Park (New South Wales, Australia).

Paired-end sequences were obtained for datasets 1 and 3 with an insert size of approximately 460 bp in order to aid in detection and assembly of indels, repetitive sequences, inversions and other genome rearrangements that would otherwise be difficult to assemble with individual short reads and no close reference [27].

We used dataset 1 to develop an NGS-based approach for *in silicio* chloroplast DNA extraction and whole chloroplast genome assembly. For comparison, and to investigate whether the longer reads from 454 assemble more effectively than Illumina reads, a whole chloroplast genome was also assembled using dataset 2. Along with dataset 1, dataset 3 was used to investigate the use of NGS for SNP detection and marker development for a study of chloroplast variability in *T. ciliata*. Sample details for each dataset can be found in Additional file 1. These methods are detailed in the following sections.

### *In silicio* chloroplast DNA assembly from whole-genome shotgun Illumina sequencing

Total genomic DNA was extracted from CTAB-preserved leaf tissue using Qiagen DNeasy Plant Mini kits. Two to three extractions per individual were pooled, concentrated in a vacuum centrifuge and quantified using the QuantiFluor dsDNA system (Promega). The detector used was a SpectraMAX Gemini XPS (Molecular Devices). The DNA was normalised and then four individuals were pooled for each of the populations in Nightcap National Park (NP) and Royal NP prior to library preparation.

Sequencing was carried out by Plant Genomics Services at Southern Cross Plant Science, Lismore, Australia. Paired-end runs (with 100 bp fragments) were performed on the Illumina Genome Analyser (GAIIx). Each sample was multiplexed along with seven samples from another experiment in one lane. Library preparation at the Plant Genomics Services was in accordance with Illumina protocol for paired-end multiplexed sequencing with minor variations. Approximately 2 μg of total DNA was sheared, polished and prepared following the manufacturer’s instructions (Illumina sample preparation protocol for paired-end multiplexed sequencing) with the following modifications. Briefly, DNA was sheared using the adaptive focused acoustics method on a Covaris S2 device for 3 mins at 6°C. Adapters were ligated to the ends of the DNA fragments involving preparation of a reaction mix on ice with a total volume of 50 μl. The reaction mixture was incubated on the thermal cycler for 15 minutes at 20°C; followed by purification using the QIAquick PCR Purification Kit and one QIAquick MinElute column, eluting in 30 μl of Qiagen EB buffer/elution buffer (EB). Additional sequences necessary for library amplification on the flow cell were added by tailed primers during the enrichment PCR also the index sequences were also added during this PCR. Ligation products were purified by 2% agarose gel electrophoresis (SizeSelect-E-Gel, Invitrogen). A narrow size range of predominantly 500 bp fragments was aspirated from the gel. After 20 cycles of PCR, products were quantified using a DNA High Sensitivity chip on an Agilent BioAnalyzer 2100. Approximately 6 pmol of pooled indexed samples per lane and 5 pmol of PhiX control were sequenced for 101x 2 cycles using version 4 Cluster and sequencing kits on an Illumina Genome Analyser (GAIIx) following the manufacturer’s instructions. Base calling was performed with Illumina software RTA v.1.6 and demultiplexing by CASAVA v.1.7 (Illumina, San Diego, CA, US).

Paired-end Illumina reads were imported into CLC bio Genomics Workbench (version 4.9, http://www.clcbio.com) (CLC). Adapters and failed reads were trimmed during import and then reads were trimmed in CLC to a quality limit of 0.05 (an error probability calculated in CLC equivalent to a minimum average Phred count of 13 for each sequence) and a minimum length of 50 bp.

Sequences obtained from the samples from northern New South Wales (NSW, dataset 1) were used for *de novo* assembly. Independent *de novo* assemblies were performed in CLC and Velvet 1.1.6. [28]. For CLC, assemblies were built with a minimum contig length of 200 bp. Conflict resolution was vote majority, match mode random, word length automatic and other parameters set to default. Settings for similarity and length fraction were 0.8 and 0.5 respectively, and a distance from 100 bp to 600 bp was selected in order to utilise paired fragments for *de novo* assembly.

For Velvet assemblies, a hash length (or k-mer length) of 83 was selected after results using hash length from all odd numbers from 71 to 93 inclusively were tested and plotted for each value against N50, maximum contig length and number of reads matched. Coverage cutoff and expected coverage were automatically selected by Velvet, and insert size is calculated internally for use in *de novo* assembly. The two independent *de novo* analyses were used to ensure overlap of contigs in order to aid assembly of the contigs into chloroplast genomes.

To isolate chloroplast sequences from the contigs, a BLASTn of each set of *de novo* contigs against a database of whole chloroplast genomes (134 seed plants downloaded from NCBI (http://www.ncbi.nlm.nih.gov/, 18 Feb 2011) was performed in CLC with default parameters. All contigs with an E-value of zero were exported to Geneious Pro v5.5.6 [29] for chloroplast genome assembly.

At the time of analysis, the closest relative to *Toona ciliata*, determined by placement of taxa within the angiosperm phylogeny [30] with a whole chloroplast genome sequence available on GenBank, was that of *Citrus sinensis* (Genbank:NC 008334; [31]). In the absence of a close reference sequence, the chloroplast genome sequence of *Citrus sinensis* was used as a scaffold for contig mapping.

To enable assembly of both inverted repeat (IRa and IRb) regions, two references were extracted from the *C. sinensis* genomes. Reference sequences had an overlap of approximately 3000 bp. One reference sequence was 117,062 bp long, began at base 1 of *C. sinensis* genome and extended across the inverted repeat b region (IRb) into the small single copy (SSC) region. The second reference was 46,172 bp long. This spanned the area from just inside the IRb (113,958 bp of *C. sinensis* genome) and included a section of *ycf*1 across the SSC up to the end of IRa. Assemblies were performed in Geneious Pro v5.5.6 (with *C. sinensis* in two parts as references) using the medium/fast sensitivity setting. The assemblies were checked and edited manually and consensus sequences from each assembly were combined to create the chloroplast genome.

Assembling with reference to the two *C. sinensis* sequences allowed for contigs that covered the IR regions to be used in both IR copies. In cases where contigs aligned only partially, searches were performed to investigate whether or not these contigs crossed the IR junctions. The four junctions between the two IRs and the SSC and large single copy (LSC) were verified by PCR amplification and Sanger sequencing. PCR primers were custom designed with Primer3 [32] and tested with Netprimer (PREMIER Biosoft International) (see Additional file 2).

PCR amplifications were performed in a CP2-03 Thermal Cycler (Corbett Research, Mortlake, Australia). PCR conditions followed those of Ebert and Peakall [33] with slight modification : each 20 uL reaction contained approximately 2 ng genomic DNA, Bioline PCR buffer, four dNTPs 0.2 um each, MgCl2 3.5 mm, forward and reverse primers 0.2um each and 0.5 U Imolase DNA polymerase (Bioline). Cycling conditions were as follows: denaturation at 94°C for 3 minutes followed by 42 cycles of 94°C for 30 s, variable annealing temperature 30s and extension at 72°C for 45 s. Annealing temperatures began at 66 and decreased by 3°C every second cycle down to 51°C, then by 4°C to 47°C for the remaining 30 cycles. Purification of the PCR products and sequencing were performed by Macrogen Corporation (South Korea). Sequences were visualised and manually edited using Geneious Pro v5.5.6 [29].

### Sub-sampling Illumina sequence data

To determine the minimum coverage required to detect and assemble the chloroplast genome of *Toona ciliata* data were sub-sampled to 50%, 25%, 12.5% of the original reads. Sub-sampling was performed on the two raw data files (forward and reverse sequences, dataset 1) using a PERL script to select every second, every fourth and every eighth sequence respectively. Using CLC with the same parameters as for the original dataset, the three new datasets were trimmed and *de novo* assemblies were performed. Chloroplast contigs were isolated using BLASTn and each contig set was assembled with the chloroplast genome constructed with the full dataset as a reference. Assembly was performed in Geneious Pro v5.5.6 with parameters as for previous assemblies.

### Chloroplast DNA isolation and assembly from 454 GS FLXII sequencing

Chloroplasts were isolated using a modified version of the method described by Jansen et al. [34]. Young *Toona ciliata* foliage was harvested and placed in the dark for 48 hours. Approximately 40 g of tissue was homogenised using a blender in 400 mL of cold isolation buffer (1.25 M NaCl 50 mM Tris–HCl pH 8.0, 5 mM EDTA, 1% BSA, 10 mM 2-mercaptoethanol, 5% polyvinylpyrrolidone). The homogenate was filtered through two layers of gauze and one layer of Miracloth (Calbiochem) and centrifuged at 1000 *g* for 15 min at 4°C. The resultant pellet was resuspended in wash buffer (10 mM Tris–HCl pH 8.0, 5 mM EDTA, 10 mM 2-mercaptoethanol, 100 μg/mL proteinase K) and distributed equally between four step gradient columns (each consisting of 18 mL of 52% w/v sucrose with an overlay of 7 ml of 32% w/v sucrose). The columns were centrifuged at 25,000 rpm for 1 hour at 4°C in an SW-28 rotor (Beckman) and chloroplast bands were removed from the 30-52% interface and pooled. The chloroplasts were then centrifuged at 3300 rpm/4°C for 15 min in a clinical centrifuge (Heraeus). This process was repeated four times, with chloroplasts being gently resuspended in 35 mL wash buffer prior to each centrifugation. The final pellet was then resuspended in 3 ml of wash buffer without added proteinase K.

Chloroplast DNA was amplified using a Repli-G whole genome amplification kit (Qiagen). 1 μL of resuspended chloroplasts was added to 4 μl phosphate-buffered saline and 1.5 μL chloroplast lysis solution (360 mM KOH, 18 mM EDTA, 90 mM DTT). The reaction was then incubated on ice for 10 min, after which 3.5 μL stop solution (Qiagen) was added. Chloroplast DNA amplification was then performed according to the Qiagen protocol using 1 μL lysis product per amplification reaction. The success of the amplification reaction and the purity of the chloroplast DNA were estimated by restriction digest as described in [34].

Sequencing on the 454 platform was performed by the Ramaciotti Centre for Analysis of Gene Function, University of New South Wales (UNSW, Australia). The chloroplast-enriched DNA was prepared for sequencing using a SPRIworks and associated kits (Beckman-Coulter). 454 sequencing was performed on 1/16 of a sequencing plate. The reads were imported into CLC and trimmed to a quality limit of 0.05 and a minimum length of 50 bp.

*De novo* assembly was performed in CLC with parameters set as for the Illumina data. The resulting contigs and a second set of contigs created by *de novo* assembly using Newbler 2.5.3 (and performed with default parameters by the Genome Sequencing Facility at UNSW) were used for chloroplast genome assembly. As for dataset 1, BLASTn (with default parameters) was performed in CLC with each of the Newbler and CLC contigs, and all contigs with an E-value of zero were used for the assembly.

The chloroplast genome for *Toona ciliata* sequenced on the 454 platform (dataset 2) was assembled in two overlapping halves using the method described above for dataset 1. Again the four IR junctions were verified by PCR amplification and Sanger sequencing.

### Verification of the chloroplast genome and SNP detection

To verify the assembly of the chloroplast genome, compare the assemblies from sequencing on the two platforms, and confirm variable SNPs, whole chloroplast genome sequences produced from datasets 1 and 2 (Illumina and 454 respectively) and the Citrus chloroplast genome from GenBank were aligned using MAFFT (online version) with default settings [35]. We then used the alignment to investigate whether differences between genomes assembled with Illumina and 454 datasets were likely to be due to SNPs or sequencing error and in what regions of the genome the SNPs occurred. Twenty-seven primer pairs were designed and used for the amplification and sequencing of IR junctions, differences between 454 and Illumina, ambiguous sequence and SNPs (Additional file 2). PCR amplifications were performed on DNA isolated using Qiagen DNeasy Plant Mini kits from each of the eight CTAB-preserved individuals used for datasets 1 and 3, as well as a fresh sample of the individual used for dataset 2.

Because DNA from four individuals was pooled for each of the Illumina shotgun sequencing datasets, we were able to detect SNPs and investigate chloroplast genome variation of *T. ciliata* within and between the two sites in Nightcap NP in northern NSW and Royal NP near Sydney. For SNP detection within and between datasets 1 and 3, trimmed reads from each sample were mapped onto the Illumina chloroplast genome using CLC with the following settings: match mode = random; conflict resolution = vote, similarity = 0.8 and length fraction = 0.9 were used for the mapping. With four individuals in each pool, frequencies of approximately 25% for each individual were expected. To determine the most appropriate settings for each dataset, SNP detection was conducted twice with minimum variant frequencies (mvf) of 10% and 20%. The threshold for false SNP calls was investigated by PCR and Sanger sequencing for every putative SNP detected at mvf = 20%, and 50% of those detected at mvf = 10%. PCR primers were custom designed with Primer3 [32] and tested with Netprimer (PREMIER Biosoft International) (see Additional file 2). Once SNPs were verified and detection parameters were optimised, SNP detection was also performed on each of the sub-sampled datasets to determine the minimum coverage of the chloroplast genome required for detection of SNPs from a pool of four individuals.

### Exploring the phylogeography of *Toona ciliata*

**Figure 1:**
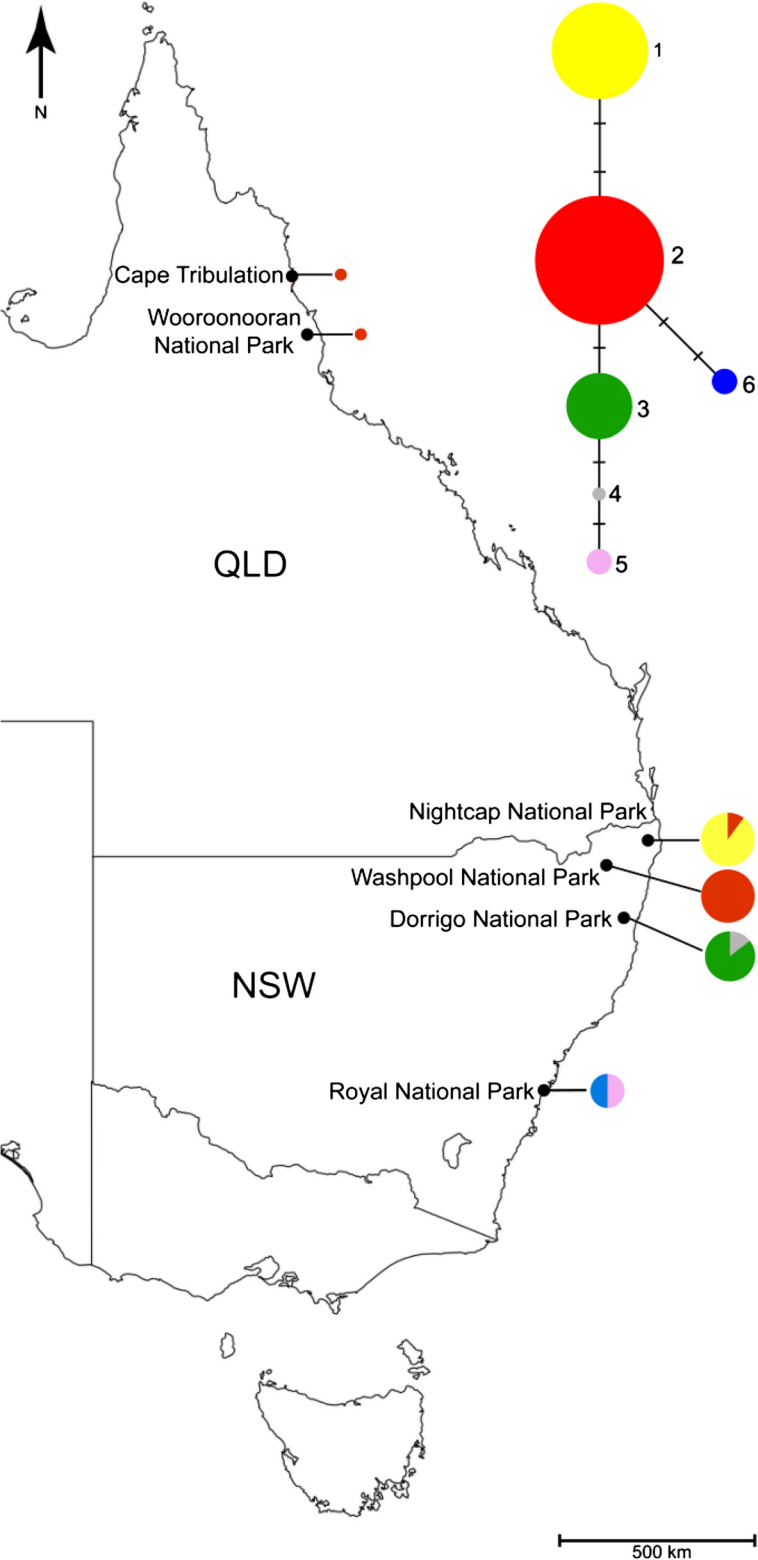
**Network and distribution of chlorotypes of *Toona ciliata *in eastern Australia as determine by NGS-derived full chloroplast analysis.** Different colours represent the different chlorotypes numbered 1–6 in the network diagram; Sizes of the circles in the network diagram are proportional to occurances of chlorotypes; Sizes of the circles on the map are proportional number of sampled individuals; QLD = Queensland; NSW = New South Wales.

*Toona ciliata* was selected for this study because of its economic and conservation significance, and because an expectation of low genetic diversity rendered it a good candidate species for testing the potential of our chloroplast sequencing technique. We compared three geographically proximate populations from northern NSW (Nightcap NP, 10 individuals; Washpool NP, nine individuals; Dorrigo NP, seven individuals) to a population from the southern distributional limit of the species (Royal NP, four individuals) and two individuals from the Wet Tropics in north-east Queensland (Figure 1). Our expectation was to find low levels of differentiation among northern NSW populations, stronger differentiation between the Queensland and NSW samples, and low diversity in the Royal NP population. Total genomic DNA was extracted from silica-preserved material using the Qiagen DNeasy Plant Mini Kit following manufacturers protocol, or by the Australian Genome Research Facility with the Nucleospin Plant II (MACHEREY-NAGEL GmbH & Co. KG) using buffer set PL2/3.

Seven of ten confirmed SNPs were amplified and sequenced for the 32 individuals (see Additional file 1 for sample details) using the same PCR conditions as for PCR verifications from previously. SNPs were used to construct chloroplast haplotypes (chlorotypes) for each individual. To visualise differences and relationships between chlorotypes, a median-joining network was constructed using NETWORK v4.6.1.0 (http://www.fluxus-engineering.com) and chlorotypes were then visualised on a map to assess whether diversity was geographically structured.

## Results

We assembled a chloroplast genome (159,514 bp long) for *Toona ciliata* from Illumina 100 bp paired-end sequencing with average coverage of 214 times (x). The length of the LSC, SSC and IR regions were found to be 87,022 bp, 18,352 bp and 27,070 bp respectively. Sanger sequencing showed that our method for assembling the chloroplast genome in two slightly overlapping sections resulted in a highly accurate assembly of the four junctions between the IRs and the SSC and LSC.

### *In silicio* chloroplast DNA assembly from whole-genome shotgun Illumina sequencing

The two *Toona ciliata* libraries (multiplexed with seven other libraries from another experiment in a lane) produced approximately 0.7 Gbp per sample. The number of reads for datasets 1 and 3 after initial filtering (using Illumina pipeline 1.5-1.7 during import into CLC) were 7,838,929 (after trimming 7,427,580) and 6,976,127 (after trimming 6,759,284) respectively with an average read length after trimming of 93 bp and 95 bp, respectively. Approximately five percent of the total number reads of reads were estimated to be chloroplast, see Table 1. Average phred scores were 31.2 for dataset one and 30.5 for dataset 3. *De novo* assembly using Velvet and CLC produced 278 and 171,603 contigs respectively (Table 1). Of these, 33 of the Velvet contigs and 12 from CLC had an E-value of zero when a BLASTn search against our whole chloroplast genome database was performed. These 55 contigs were used for whole genome assembly.

**Table 1:** Summary of de novo assemblies and BLASTn results for Illumina and 454 genomes

	**Illumina (159,514) bp)**		**454 (159,382 bp)**	
	**CLC**	**Velvet**	**CLC**	**Newbler**
Total number of contigs	171 603	278	126	39
Mean contig length	261	769	1 565	5 518
Longest contig	49 987	13 879	20 405	33 064
N50	252	3 049	4 065	18 420
Number of chloroplast contigs (E value = 0)*	12	33	24	11
Longest chloroplast contig	33 886	12 240	20 405	33 060
Sum (bp) of chloroplast contigs	146 401	129 686	132 317	132 215
Chloroplast reads (% of total reads) **	5.25%		99.00%	

Genbank accession numbers for the 80 contigs GenBank:JX445406 – Jx445485.

*determined by a BLASTn search against a database of 134 whole chloroplast genomes downloaded from GenBank.

**determined by mapping the total reads onto the whole chloroplast genome for *Toona ciliata* in CLC with default settings.

The sum of the chloroplast contigs produced by CLC and Velvet were 146,401 bp and 129,686 bp respectively (Table 1). Eighty-six percent of the total number of base pairs contained in the contigs from the two approaches was congruent. Contigs created in the two programs aligned with five mismatches, and no inversions or deletions. The five mismatches were all at positions with coverage of at least three contigs and the mismatch occurred among contigs assembled with the same program. In five cases the last 5–25 bases of a contig did not align. It is possible these areas could have been eliminated with more stringent trimming, however, since these also occurred in areas covered by at least three contigs and the others aligned without mismatches, these bases were discarded from the assembly. Contigs from two independent read assemblers combined to produce a partially overlapping, complementary contig map to the *Citrus sinensis* scaffold. Assembly of the contigs with no reference at all produced the same consensus, however to capture the IR junctions correctly required more manual editing. The only region found with no overlapping contigs was tested by alignment with the 454 chloroplast genome and Sanger sequencing and it was confirmed that the contigs were correctly assembled adjacent to one another against the *Citrus sinensis* scaffold, but with a zero length gap in the *T. ciliata* chloroplast consensus sequence.

### Sub-sampling Illumina sequence data

Sub-sampling dataset 1 resulted in an average coverage of 102.06 x with 50% of the total number of reads, 50.96 x with 25% or reads and 25.36 x with 12.5% of the reads. With 50% and 25% of the original reads, the chloroplast contigs from the *de novo* assembly covered the SSC, LSC and IR without gaps, however with 12.5% of the reads it was no longer possible to construct the full chloroplast genome.

### Verification of the chloroplast genomes and comparison between Illumina and 454 assemblies

The 454 sequencing of *Toona ciliata* chloroplast DNA produced 85,119 reads (84,104 reads after trimming) with an average read length of 329.5 bp. The average coverage was 170 x and 99% of the total number of reads were estimated to be chloroplast (see Table 1). The 454 chloroplast genome was 159,382 bp long, 132 bp shorter than that produced by Illumina sequencing. Sanger sequencing confirmed that the junctions between the IRs and LSC and SSC were assembled correctly with both platforms. Alignment of the 454 and Illumina chloroplasts showed 15 differences between the Illumina and 454 sequences. Sanger sequencing showed that four differences were due to real SNPs and two gaps in the 454 sequence were 454 sequencing errors. A 28 bp gap in the IRa near the junction with the SSC was incorrect in the Illumina assembly. The remaining eight positions were homopolymer repeats of either A or T and were, in all cases, correctly sequenced with Illumina rather than 454.

### SNP detection and PCR verification

Pooling of individual samples for the library preparation enabled us to develop phylogeographically informative SNP markers. Furthermore, with two shotgun sequencing libraries (datasets 1 and 3) available we were able to investigate within and between site variation by mapping the reads for each site onto the Illumina chloroplast genome. Mapping of the reads showed average coverage of 214 x and 208 x for datasets 1 and 3, respectively. The number of putative SNPs detected in reads of dataset 1 was 30 with a minimum variant frequency (mvf) of 10%, and 3 with mvf of 20%. For dataset 3, 49 and 20 putative SNPs, respectively, were detected at the same mvf levels. We estimated, by visual examination of the read mapping, that ten of the putative SNPs were likely to be real. The remaining SNP calls were in areas of low coverage and/or poor alignment. Sanger sequencing confirmed that none of the SNPs detected at a minimum variant frequency of 10-20% was real. Of the 20 SNPs detected at the 20% threshold, the ten SNPs we predicted by eye were confirmed as real. Thus, visual examination of the read mapping would have been sufficient for selecting SNPs. Once confirmed we optimised our SNP detection approach by increasing stringency of mapping in CLC to include a length fraction of 0.8 and similarity 0.9. Subsequent SNP detection with mvf 20 and minimum coverage of 10 resulted in retrieval of only the ten real SNPs. None of the SNPs were found in regions that are amplified using universal sequencing primers for phylogeographic studies, for instance, three of the SNPs were within coding regions. No fixed differences between datasets 1 and 3 were found. Sanger sequencing of the eight individuals (four each, pooled into datasets 1 and 3) revealed no further SNP variants. Overall, the relative frequencies of the variants detected by NGS did not correspond with the frequencies found by Sanger sequencing.

When SNP detection was performed on the sub-sampled data, using the optimised parameters outlined above, all ten SNPs were detected with 50% of the reads. With 25% of the reads nine of the ten were retrieved. Using an eighth of the data the SNPs called were not reliable.

All sequences generated during chloroplast genome verification, comparison of 454 and Illumina assemblies, SNP detection and verification have been lodged in the GenBank database at NCBI [GenBank: JX445486-JX445910].

### Exploring the phylogeography of *Toona ciliata*

Seven of the ten confirmed SNPs were used for a phylogeographic study of *Toona ciliata.* Two SNPs in homopolymer repeat regions were excluded from the network analysis and a consistent unambiguous sequence could not be obtained for the remaining one. The combination of seven chloroplast SNPs produced six chlorotypes across five populations (Figure 1, Additional file 1)*.* Excluding the Queensland samples, Washpool NP was the only population with only one chlorotype; two chlorotypes were found at the other three NSW sites. Chlorotype 2, the most common chlorotype in the network, was shared across three geographic regions, the Wet Tropics in far north Queensland, Washpool NP, and Nightcap NP (one individual). All other chlorotypes were population-specific, showing geographic structure (network in Figure 1). The network suggests that the two chlorotypes from Royal NP originate from two distinct lineages.

## Discussion

### *In silicio* chloroplast DNA assembly from whole-genome shotgun Illumina sequencing

We have developed a fast and efficient approach for obtaining whole chloroplast genome sequences and detecting chloroplast variation. We successfully assembled a complete chloroplast genome sequence of *Toona ciliata* without a close reference genome using shotgun sequencing of whole genomic DNA on the Illumina platform.

The new chloroplast genome was used to develop SNP markers for screening diversity in *T. ciliata*. The preliminary phylogeographic study showed that seven of the identified variable regions were informative for a study of this species. Morris *et al*. [36] highlight that as with many traditional (Sanger) sequencing based phylogeographic studies, an NGS approach such as this does not overcome the possibility of ascertainment bias. Since our SNP discovery panel was based on only two populations it is possible that we have missed SNPs across the distributional range of the species. As technological advances bring greater coverage and potential for multiplexing it will be possible to broaden the sampling for SNP discovery, thereby reducing bias. This approach will then be increasingly useful for landscape-level studies.

We used Sanger sequencing to validate our NGS data, including sequencing of the inverted repeat junctions, regions of ambiguous sequence, SNPs, and differences in sequence between Illumina and 454 approaches. Verification sequences, along with the two independent *de novo* assemblies, confirmed that our method for constructing chloroplast genomes yields high quality sequence that correctly assembles across the inverted repeat junctions. We also optimised mapping parameters for SNP detections and confirmed that the 20% minimum variant threshold for detecting SNPs in pooled samples of four individuals was appropriate. No further variants were detected with Sanger sequencing of the seven loci across 32 individuals, indicating that NGS sequencing of pooled individuals captured all the variation present in the samples. Although some verification by PCR and sequencing may be necessary for assembly of further chloroplast genomes by this method, the level required should be far less intensive, particularly considering that with optimised parameter settings automatic SNP detection was reliable. Furthermore, for molecular ecology studies exploring chloroplast variation, it is not necessary to assemble whole chloroplast genomes.

Our study has shown that there is very little difference between the quantity and quality of chloroplast DNA sequenced by Illumina shotgun sequencing compared with 454 sequencing of enriched chloroplast DNA. It is well documented in the literature that 454 platforms have a higher rate of error sequencing homopolymer repeats than Illumina due to differences in sequencing technology [37-40]. As expected, we found that neither sequencing platform was consistently reliable for reading homopolymer repeat regions, although Illumina outperformed 454. Since we did not denoise our 454 data some of the errors may have been exaggerated [41]. Nevertheless, variable regions were detected and markers could be developed to investigate these regions further if required.

Given that extraction of chloroplast DNA from a wide range of rainforest tree species is time consuming and would require optimisation across a range of taxa, this method using whole genomic DNA, sequenced on the Illumina platform, is preferable for large scale comparative studies of plants. Sequencing output is constantly increasing, thereby increasing opportunities for larger multiplexed experiments, bearing in mind that genome sizes of plants vary and if not considered this will effect the coverage and ultimately chloroplast assembly.

Morris *et al.*[36] present an alternative approach, shallow sequencing samples to detect SNPs across the *Panicum virgatum* genome. There are also many genome reduction methods (see Cronn *et al.*[18] for a review of current approaches) that can be applied to molecular ecology studies of this kind. At present for large landscape scale studies the choice of the most effective approach is limited by multiplexing ability and cost. Our approach is a cost-effective and bioinformatically simple method for a study of this kind.

### Exploring the phylogeography of *Toona ciliata*

*Toona ciliata* contains very little chloroplast variation across its distributional range. Thus, many commonly used markers would probably not uncover informative variation. However, using a whole chloroplast genome approach we were able to elucidate some landscape-level patterns in *Toona ciliata.* This approach has great potential for single as well as multi-species studies.

Overall, the low levels of chloroplast diversity observed in *Toona ciliata* support the possibility of a founder event either through recent colonisation of Australia from the north, or an extreme bottleneck followed by rapid expansion. However, although only one individual was sampled from each of two Wet Tropics sites, finding a single chlorotype shared with NSW was unexpected given that these areas are geographically distant, and that the Wet Tropics are in closer proximity to the extensive Indo-Malesian distribution of the species (Figure 1). The frequency and position of chlorotype 2 on the network is in agreement with a possible ancestral origin, not conflicting with a recent north to south colonisation path. With greater sampling this may be explored further. The strong differentiation between Nightcap and Dorrigo was also unexpected given their close proximity and shared species assemblages, as was the diversity detected in Royal NP. However, areas of Royal NP have been replanted with rainforest trees, and although sampling was aimed at avoiding planted individuals, mixed provenance cannot be excluded.

## Conclusions

A simple approach for detecting chloroplast variation from Illumina shotgun sequencing of whole genomic DNA from pooled individuals would include the following steps:

1. *De novo* assemble the trimmed reads by two independent methods.

2. BLASTn against a chloroplast genome database and retain contigs with an E-value of zero (more can be used if the reference is distantly related).

3. Assemble contigs with reference to chloroplast genome of the closest available relative, assembling in two halves if both IR regions are required.

4. Use consensus chloroplast sequence for mapping reads and detecting SNPs.

The technical approach presented here provides a simple method for construction of whole chloroplast genomes from shotgun sequencing of whole genomic DNA using short-read data and no closely related reference genome. The high coverage of Illumina sequence data also renders this method appropriate for multiplexing and SNP discovery and therefore a useful approach for landscape level studies of evolutionary ecology, particularly given that Illumina sequencing is the most cost effective platform presently available [21].

We have shown by sub-sampling dataset 1 (to 25% of the original number of reads) that approximately 0.18 Gbp of shotgun Illumina data would have been sufficient for assembly of the whole chloroplast genome of *Toona ciliata*. With current Illumina HiSeq output at approximately 35 Gbp per lane there is already the potential for producing up to 120 chloroplast genomes per lane by this method (depending on genome size and required sequencing coverage). The present limitation is the cost and availability of labels for multiplexing (there are 96 unique labels currently commercially available). As library costs decrease, we will see even more opportunities for discovering variable loci for large numbers of species at low cost, or for directly comparing chloroplast DNA from multiple pooled populations. We are currently exploring the latter approach to identify common phylogeographic patterns among a range of co-distributed rainforest trees.

### Availability of supporting data

The data sets supporting the results of this article are available in the GenBank repository as follows:

All 80 contigs generated by this study and used for assembly of the *Toona ciliata* chloroplast genome have been lodged in the GenBank database at NCBI [GenBank:JX445406-JX445485].

All sequences generated during chloroplast genome verification, comparison of 454 and Illumina assemblies, SNP detection and verification have been lodged in the GenBank database at NCBI [GenBank:JX445486-JX445910].

## Competing interests

The authors declare that they have no competing interests.

## Authors’ contributions

HM participated in the design of the study, contributed molecular work for the Illumina component, participated in sequence alignment, bioinformatic analyses and drafted the manuscript; MvdM participated in the design of the study, sequence alignment, bioinformatic analyses and helped to draft the manuscript; SKD contributed molecular work for the 454 component and helped to draft the manuscript; MAE performed library preparation and sequencing for the Illumina component, provided the subsampling script, assisted with bioinformatic analyses and provided comments on the draft manuscript; RJH participated in the design of the study, and provided advice on molecular and bioinformatic components; EM participated in bioinformatic analyses and optimizing technical approach; PDR contributed molecular work for the Illumina component, participated in bioinformatic analyses and provided comments on the draft manuscript; MLM performed PCR and sequence alignment for verification and phylogeographic components, performed the phylogeographic analysis and helped to draft the manuscript; JS participated in sequence alignment and optimizing technical approach; MR conceived of the study, and participated in its design and coordination and helped to draft the manuscript. All authors read and approved the final manuscript.

## Supplementary Material

Additional file 1 Toona ciliata samples used for this study. Click here for file

Additional file 2 PCR primers designed for SNP detection, assessment of differences between Illumina and 454 sequencing and verification of inverted repeat flanking regions in Toona ciliata. Click here for file
